# A three-week mindfulness intervention on mental skills, internal-load regulation, and performance in youth swimmers: a randomized controlled trial

**DOI:** 10.1038/s41598-026-48457-8

**Published:** 2026-04-14

**Authors:** Mohamed Ali Sifi, Hamza Marzouki, Okba Selmi, Bilel Sdira, Bilel Cherni, John Elvis Hagan, Dan Iulian Alexe, Alexandru Ioan Băltean, Vlad Adrian Geantă, Wafa Douzi, Anissa Bouassida

**Affiliations:** 1 High Institute of Sport and Physical Education of Ksar-Said, University of Manouba, 2010 Manouba, Tunisia; 2 High Institute of Sport and Physical Education of Kef, University of Jendouba, 7100 Kef, Tunisia; 3 Research Unit: Sport sciences, Health and Movement, University of Jendouba, 7100 Kef, Tunisia; 4 Department of Health, Physical Education and Recreation, University of Cape Coast, Cape 24 Coast PMB TF0494, Cape Coast, Ghana; 5 Neurocognition and Action - Biomechanics Research Group, Faculty of Psychology and Sports Science, Bielefeld University, Bielefeld, Germany; 6 Department of Physical and Occupational Therapy, “Vasile Alecsandri” University of Bacau, 600115 Bacau, Romania; 7 Department of Physical Education and Sport, Faculty of Physical Education and Sport, Aurel Vlaicu University of Arad, 310330 Arad, Romania; 8 Laboratory « Mobilité, Vieillissement, Exercice » (MOVE) - UR 20296, Faculty of Sports Sciences, University of Poitiers, 8 Allée Jean Monnet, Poitiers, 86000 France; 9 Department of Business Economics, Health and Social Care, University of Applied Sciences and Arts of Southern Switzerland, Landquart, Manno, Switzerland; 10 University College Physiotherapy Thim van der Laan, Landquart, GR Switzerland

**Keywords:** Psychological resilience, Self-regulation, Perceived exertion, Heart rate, Swimming endurance, Cognitive skills, Health care, Neuroscience, Physiology, Psychology, Psychology

## Abstract

This study examined whether a three-week mindfulness program integrated into swim training could enhance mental skills, stabilize internal-load responses, and affect short-term endurance performance in youth swimmers. Thirty trained swimmers (20 males, 10 females; aged 19.7 ± 1.7 years) were randomly assigned to an experimental group (EG, *n* = 15) or a control group (CG, *n* = 15). The EG completed 12 pre-swim mindfulness sessions, each lasting 30–45 min and incorporating breathing regulation, body awareness, and attentional-focus exercises, while the CG followed standard training only. Pre- and post-intervention assessments included 400 m freestyle time, mean velocity, and mental skills (basic, psychosomatic and cognitive). Peak heart rate (HRpeak), expressed as a percentage of theoretical maximum HR, was recorded throughout each training session, and the rate of perceived exertion (RPE) was assessed at the end of each session. The EG exhibited greater improvements in mental skills across basic, psychosomatic, and cognitive domains (all *p* < 0.01; cohen’s d = 2.02–2.83) compared with the CG. Despite higher HRpeak and RPE values (*p* < 0.001), the EG showed significantly lower intra-subject and inter-session variability, indicating a more stable internal-load profile. No significant interactions were observed for 400-m performance variables (*p* > 0.05). Mindfulness appears to improve athletes’ self-regulation and training consistency rather than producing immediate performance gains. Longer interventions are warranted to evaluate the long-term transfer of these benefits to competition settings.

**Supplementary Information:**

The online version contains supplementary material available at 10.1038/s41598-026-48457-8.

## Introduction

Mindfulness-based approaches are increasingly applied in sport to strengthen athletes’ capacity for attention control, emotion regulation, and self-awareness, key components of self-regulation that underpin consistent performance under stress^1,2^. By enhancing focus on present-moment experience and reducing cognitive distraction, mindfulness may influence both physiological responses, such as heart rate (HR) and perceived exertion (RPE), and behavioral outcomes, such as pacing and technique^3–5^. Evidence from different athletic settings shows that mindfulness training enhances psychological skills and training quality, yet its effects on physical or performance indicators remain inconsistent and appear sensitive to context, intervention duration, and sport characteristics^6–8^.

Swimming provides a particularly suitable model for investigating mindfulness in sport. As a closed-skill, pacing-dependent activity performed in a sensory-restricted environment, swimming requires sustained internal focus and precise self-regulation of effort. Limited sensory feedback, constrained breathing, and repetitive movement patterns make swimmers highly dependent on interoceptive awareness to maintain optimal pacing and technique^9,10^. Under these conditions, even small lapses in attention or emotion control can translate into measurable fluctuations in physiological and perceptual load, which can be systematically tracked through HR and RPE monitoring across training sessions^5,11^. Embedding mindfulness within swimming sessions therefore offers a practical framework to examine whether enhanced attentional and emotional regulation can stabilize internal-load responses and support consistent training execution.

Despite the growing body of research on mindfulness in sport, studies conducted in aquatic disciplines remain limited. Previous interventions lasting four to eight weeks have reported improvements in attentional control, anxiety management, and overall psychological readiness in swimmers, but performance outcomes were inconsistent or modest^7,8,12^. In contrast, emerging evidence from cognitive neuroscience and applied sport psychology indicates that shorter mindfulness exposures can already trigger measurable changes in mental and physiological states. For example, Tang et al.^13^ demonstrated improved attention and self-regulation after only five days of meditation, while Sondt et al.^14^ observed reduced stress-recovery imbalance following a ten-day intervention in athletes. These findings support the feasibility of brief, in-season mindfulness cycles designed to complement physical training without disrupting competition schedules.

Within this framework, the present study examined whether integrating a three-week mindfulness program into routine swim training could enhance mental skills and stabilize internal-load responses, and whether these adaptations might extend to short-term endurance performance. It was hypothesized that swimmers exposed to mindfulness training would demonstrate greater improvements in psychological skills and more consistent internal-load regulation compared with controls, whereas no substantial differences were expected in performance over such a short duration.

## Materials and methods

### Participants’ selection

**Fig. 1:**
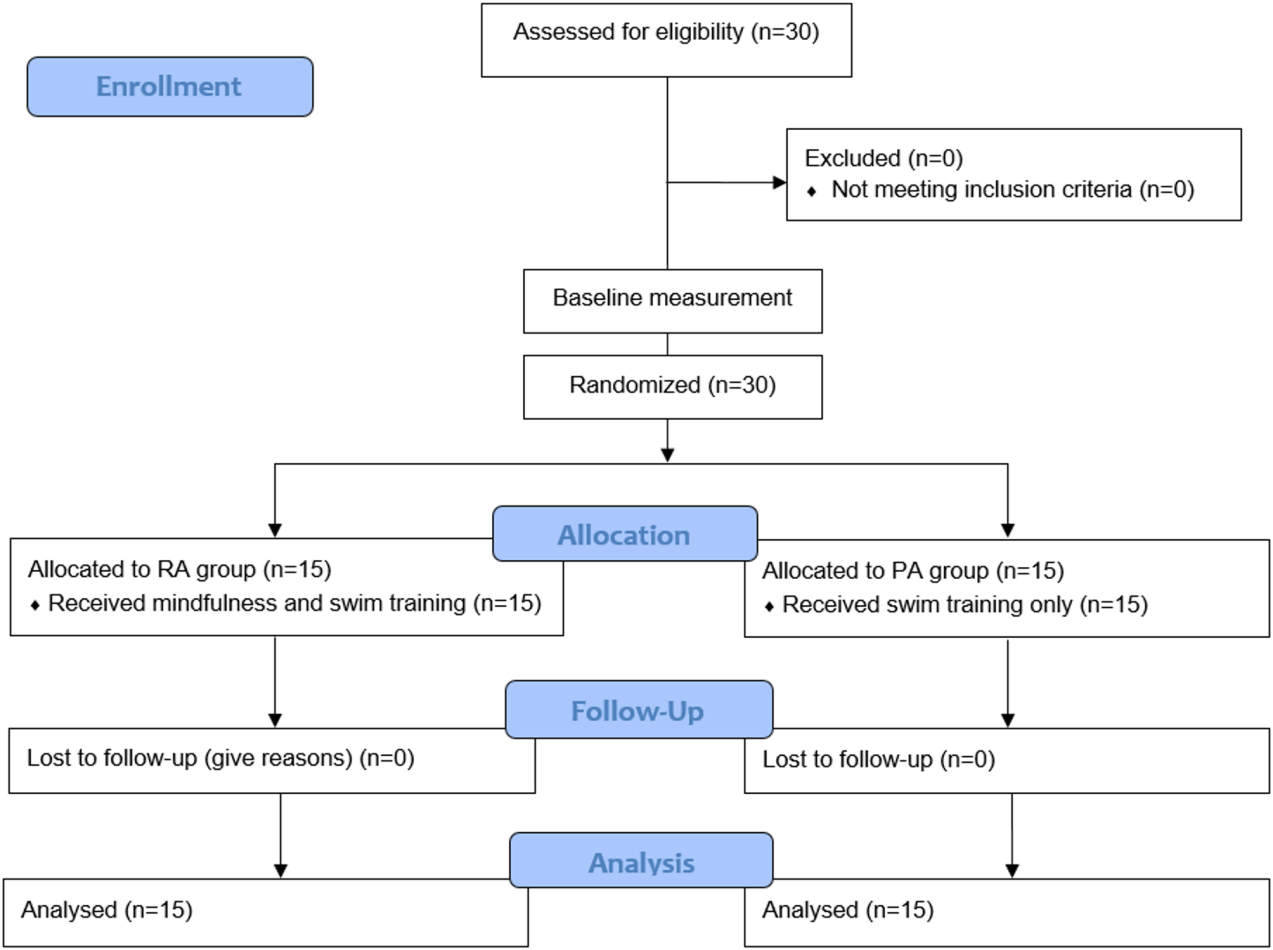
CONSORT (Consolidated standards of reporting trials) Flow diagram of the progress through the phases of a randomized trial of two groups.

Prior to recruitment, a sample size estimation was performed using G*Power software (version 3.1.9.4, University of Kiel, Germany) based on analysis of variance (ANOVA) with repeated measures and within-between interaction^15^. With α = 0.05, power (1 - β) = 0.80, and an expected medium-to-large interaction effect (f = 0.30)^13,16^, the minimum required sample was 24 participants (12 per group). To address potential dropout risks, 30 trained youth swimmers (20 males, 10 females) from the same club volunteered to participate. Randomization was performed by an independent researcher using a computer-generated sequence (permuted blocks stratified by pre-test 400-m freestyle performance). After baseline testing, participant identifiers were coded and managed by a blinded assessor not involved in testing or training. Group assignments were communicated by the independent researcher only after enrollment was completed, ensuring allocation concealment. Participants were divided into two groups of equal size (EG and CG, *n* = 15 each; 10 males and 5 females). The Participants’ characteristics are presented in Table 1. The EG received mindfulness training before swimming sessions, while the CG followed standard swim routines. Inclusion criteria were: (a) a minimum of two years of continuous swim training; (b) no severe musculoskeletal injuries within the past year; (c) no mild to moderate injuries within the past month; and (d) no prior experience with mindfulness programs. Only participants who completed at least 90% of all training and testing sessions were included in the final analysis. Figure 1 presents the CONSORT flow diagram of the study. All participants maintained their usual diet and refrained from additional fitness/dietary practices. All participants regularly competed at the national level and followed an in-season training microcycle of four weekly sessions (65–95 min each) with no competitions during the intervention. Written informed consent was obtained from all participants, and for minors, parental consent was also secured. The research protocol was approved by the local institutional research ethics committee and conducted in accordance with the Declaration of Helsinki.

**Table 1:** Participants’ demographic, anthropometric and physiological characteristics.

	Age (years)	Height (cm)	Body mass (kg)	BMI (kg.m^− 2^)	HRmaxTheo (bpm)
EG (*n* = 15)	19.4 ± 1.8	173.3 ± 7.2	66.5 ± 9.8	22.1 ± 2.5	194.4 ± 1.2
CG (*n* = 15)	19.7 ± 1.7	175.1 ± 6.7	73.8 ± 7.0†	24.0 ± 1.2†	194.2 ± 1.2

The values are expressed as mean and SD. BMI: body mass index; HRmaxTheo: theoretical maximum heart rate; EG: experimental group; CG: control group.

† Significantly different from EG at pre-test.

### Experimental design

The present investigation was a parallel-group, randomized controlled trial with a pre-to-post testing design. It aimed to test whether a three-week mindfulness program integrated into swim training could enhance mental skills, stabilize internal load, and affect 400 m freestyle performance in youth swimmers. Participants were randomly assigned to either an EG or a CG. The EG engaged in a mindfulness training program (four sessions per week) prior to their standard swimming routines, while the CG followed their usual swim training routine only and did not receive any mindfulness instruction, psychological strategy, or alternative pre-session activity. Both groups completed the same swimming training program in terms of content, volume, and prescribed intensity, and the only between-group difference was the addition of the mindfulness component before training in the EG. The intervention protocol was maintained throughout the study to meet the assumptions of a randomized controlled design.

One week prior to the intervention, all participants completed two familiarization sessions (~ 48 h apart) to ensure competence with testing and training procedures. This included acclimatization to HR monitor usage (Polar Team Pro, Kempele, Finland) and instruction in the Borg Rating of Perceived Exertion Scale (0–10 arbitrary units [AU])^5^. During this period, anthropometric measurements were taken: body mass was recorded to the nearest 0.1 kg using a digital scale (OHAUS, Florham Park, NJ, USA), height to the nearest 0.01 m, and body mass index (BMI) was subsequently calculated.

The study lasted five weeks: one week for baseline testing, three weeks for the intervention, and one week for post-testing. It was conducted during the in-season period (January-March 2021) under standardized pool and ambient temperatures (28–29 °C and 27–28 °C, respectively). Pre- and post-intervention tests included the 400-m freestyle and OMSAT-3 mental skills assessments. To minimize fatigue-related confounding, all testing sessions were scheduled approximately 48 h after participants’ last competition or high-intensity training session. Tests were performed under standardized conditions between 17:30 and 20:30, matching participants’ habitual training times. On the first evening, participants completed the 400 m freestyle test, with strong verbal encouragement. Swimmers were instructed to refrain from consuming ergogenic drinks or food for at least two hours before testing and to avoid strenuous activity the preceding day. They were also advised to maintain consistent training, sleep, and dietary routines throughout the study.

On the second testing evening, 30 min before the training session, the Ottawa Mental Skills Assessment Tool (OMSAT-3) was administered to evaluate mental skills. Efforts were made to manage blinding across all assessments by coding participants for evaluator blinding and by administering all tests, including the OMSAT-3, under standardized conditions with identical instructions and timing for both groups. The intervention was conducted in a 25-m pool with a depth ranging from 1.50 to 2.50 m.

Throughout the intervention, exercise intensity and internal load responses were monitored. HR was continuously recorded during each session and expressed as a percentage of the theoretical maximum HR (%HRmaxTheo). Subjective exertion was assessed using RPE, administered three minutes after each session to reflect perceived effort and fatigue.

### Procedures

#### Endurance 400 m front crawl

The 400 m front crawl test is a widely recognized method for evaluating aerobic endurance, technical efficiency, and physiological responses in youth swimmers. It is designed to replicate the demands of middle-distance competitive swimming. Each testing session began with a standardized warm-up consisting of approximately 1000 m of freestyle swimming at low to moderate intensity, subjectively selected by the swimmers based on their perceived exertion. This was immediately followed by a 400 m freestyle swim performed at maximal effort. To control for competitive effects, swimmers were paired based on skill level, as determined by a pre-established coach ranking. The order of races was randomized using a random number generator to eliminate potential order effects. Race starts were audibly signaled by the coach, and performance times (in seconds) were recorded by two independent observers using SEIKO S120-4030 stopwatches (Tokyo, Japan). The average of the two recorded times was used for further statistical analysis, with timing initiated the moment the swimmer’s feet left the starting block. Mean swimming velocity was also calculated for each swimmer as part of the performance analysis. After completing the test, all participants underwent a cool-down session to facilitate recovery and minimize injury risk.

#### Heart rate response

Heart rate was continuously monitored during each swimming session using a water-resistant HR monitoring system (Polar Team Pro, Polar^®^, Kempele, Finland). Recovery periods were excluded from the analysis to focus specifically on HRpeak defined as the highest value recorded during the training session. To standardize exercise intensity across groups, HR values were expressed as a percentage of HRmaxTheo (%HRmaxTheo), calculated using the formula: HRmaxTheo = 208 - (0.7 × age)^17^.

#### Rating of perceived exertion

Following each training session, swimmers subjectively rated their perceived exertion using Borg’s 10-point Likert scale (0–10 AU), with 0 corresponding to ‘no exertion’ effort and 10 to ‘maximal exertion’^5^. RPE was collected three minutes after each session to integrate both physical and psychological aspects of effort perception.

#### Mental skills measurement

Three domains of mental skills were assessed using the OMSAT-3^18^, culturally and linguistically validated for the target population. The tool evaluates twelve mental skills categorized into three domains:

- Basic skills: goal setting, self-confidence, and commitment.
- Psychosomatic skills: stress reactions, fear control, relaxation, and activation.
- Cognitive skills: imagery, mental practice, control of distractions, competition planning, and self-assessment.

Each skill was rated on a 7-point Likert scale, offering a comprehensive profile of athletes’ self-perceived psychological readiness and mental performance capabilities. The OMSAT-3 has shown high internal consistency (Cronbach’s α = 0.81–0.91) and factorial validity^18^. Questionnaires were administered under identical standardized conditions by trained researchers blinded to group allocation.

### Combined mindfulness and swimming training program

A 12-session integrated mindfulness and swimming training program was implemented to enhance both psychological resilience and physical performance. A detailed overview of the program is presented in Table 2. Each session began with a 30-45-minute mindfulness component delivered before the swimming session. Rather than directly reproducing a fully standardized framework such as Mindfulness-Based Stress Reduction or Mindful Sport Performance Enhancement, the intervention was designed as a study-specific program informed by mindfulness-based principles and sport-applied recommendations from previous literature^19,20^. These sources guided the selection of core elements, including present-moment attentional focus, breathing regulation, body awareness, and emotion-regulation strategies, which were adapted to the swimming context and the practical constraints of the study. The mindfulness component was delivered by a certified mental training instructor independent of the research team to minimize expectancy bias. Sessions emphasized present-moment awareness, emotional regulation, and attentional control through diaphragmatic breathing, box breathing, and body-scan meditation. More specifically, the mindfulness intervention was delivered immediately before each swimming session, in a quiet space adjacent to the pool, to the entire EG simultaneously by the same certified instructor. This procedure was standardized across the 12 sessions to ensure consistency of delivery. Attendance was recorded at each session to ensure adherence.

**Table 2:** Description of the combined mindfulness and swimming training program.

Sessions	Mindfulness component (30–45 min)	Swimming component (65–90 min)						
		Main objective	Warm-Up	Main set	% Theoretical HRmax	Cool down	Total distance	Times
1	Introduction to Mindfulness; 5-min guided breathing	Basic Endurance	400 m freestyle, 200 m kick, 200 m drills	4 × 200 m + 8 × 100 m freestyle, 20s/15s rest, moderate pace	75–85%	200 m easy freestyle	2600 m	75 min
2	Body Scan Meditation; Progressive muscle relaxation	Breathing Technique & Coordination	400 m mixed, 200 m arm drills	20 × 50 m bilateral breathing + 400 m moderate pace	65–75%	200 m easy backstroke	2200 m	65 min
3	Mindful Breathing; Diaphragmatic and box breathing	Anaerobic Threshold Training	400 m freestyle, 4 × 50 m build-up	4 × 300 m threshold pace, 30s rest	85–90%	200 m easy breaststroke	2000 m	70 min
4	Mindful Walking; Applied to pool deck walking	Sprint & Maximal Speed	400 m freestyle, 200 m kick	16 × 25 m sprint + 8 × 75 m variable pace	90–95%	400 m easy choice	2000 m	70 min
5	Mindful Eating; Focus on pre-training nutrition	Negative Split & Race Management	400 m freestyle, 200 m drills	3 × 400 m negative split + 4 × 100 m build-up pace	85–90%	300 m mixed easy swim	2500 m	75 min
6	Thought Observation; Applied during technique drills	Technique & Core Stability	300 m freestyle, 300 m drills	16 × 75 m (25 m drill + 50 m technique focus) + 200 m fluid swim	65–75%	200 m easy freestyle	2200 m	65 min
7	Emotional Awareness; RAIN technique pre-workout	Advanced Aerobic Capacity	500 m freestyle, 300 m pull buoy	4 × 500 m strong aerobic pace, 30s rest	75–85%	200 m easy backstroke	3000 m	90 min
8	Loving-Kindness Meditation; Team cohesion focus	400 m Race Simulation	400 m mixed, 200 m kick	2 × 400 m race pace + 8 × 50 m sprint	90–95%	400 m easy swim	2200 m	75 min
9	Mindfulness in Daily Life; Pool routine mindfulness	Descending Pace Series	400 m freestyle, 200 m IM	4 × 200 m descending + 4 × 100 m fast	85–95%	200 m easy breaststroke	2000 m	75 min
10	Stress Reduction Techniques ; Pre-race anxiety focus	Technique : Crawl Correction & Efficiency	300 m freestyle, 300 m drills	4 × 100 m (technique focus) + 8 × 50 m single-arm freestyle + 4 × 50 m fingertip drag drill + 4 × 25 m sculling + 2 × 50 m catch-up drill	65–75%	200 m mixed easy swim	2000 m	65 min
11	Mindful Communication ; Coach-athlete interaction	Endurance & Speed Combination	500 m freestyle, 200 m kick	5 × 200 m high pace + 5 × 100 m race pace	85–90%	300 m mixed easy swim	2500 m	80 min
12	Review and Future Practice; Competition mindset	Pre-Competition Taper	400 m mixed, 4 × 100 m build-up	4 × 100 m race pace + 4 × 50 m maximal speed	90–95%	400 m easy swim	1800 m	65 min

To improve reproducibility, each mindfulness session followed the same general sequence, while the specific content progressed from introductory awareness practices to more applied self-regulation strategies. Each session typically included: (i) a brief introductory phase (~ 3–5 min), during which the instructor presented the objective of the session and guided participants into a stable and comfortable posture; (ii) a guided mindfulness practice (~ 10–15 min) centered on the theme of the day; (iii) an applied awareness phase (~ 10–15 min), during which swimmers were encouraged to notice breathing rhythm, bodily sensations, muscle tension, emotional state, and distracting thoughts in a non-judgmental manner; and (iv) a short transfer phase (~ 5–10 min), during which the instructor explicitly connected the mindfulness content to the upcoming swimming session (e.g., breath control, pacing awareness, attentional refocusing, emotional regulation, or pre-performance readiness).

The progression of the intervention was organized from basic to more applied mindfulness skills. Early sessions focused on introductory practices such as guided breathing and body-scan meditation to develop present-moment awareness and attentional anchoring. Intermediate sessions introduced diaphragmatic breathing, box breathing, mindful walking, mindful eating, and thought observation, with the aim of strengthening interoceptive awareness and attentional control. Later sessions emphasized emotional awareness, stress reduction, loving-kindness meditation, mindful communication, and competition mindset, in order to help swimmers manage activation, remain focused under training demands, and improve psychological readiness before demanding sets. Although each session had a distinct theme, all sessions were based on the same core principles of present-moment attention, non-judgmental observation, and deliberate redirection of attention toward task-relevant cues.

Following mindfulness practice, swimmers transitioned into a 65-90-minute swimming module targeting 400-m freestyle performance. Both groups completed identical swim training volumes and intensities (65–95% HRmaxTheo)^3,10,21^, with the only difference being the pre-session mindfulness intervention in EG. The sessions were periodized to progressively develop aerobic capacity, pacing control, and technical efficiency (Table 2). Thus, the study design allowed the effect of the mindfulness component to be examined without altering the swimming load, content, or progression performed by the two groups.

### Statistical analysis

All data are expressed as mean ± standard deviation (SD) and 95% confidence interval (95% CI). The Shapiro-Wilk test was used to assess normality, and Levene’s test was applied to evaluate the homogeneity of variances. Variables that were not normally distributed (e.g., 400 m performance, mean velocity, and basic skills) were log-transformed prior to analysis. Intra-subject and inter-session variability of HRpeak and RPE were computed as coefficients of variation (CV%) with 95% CIs^22^. Lower CV% values were interpreted as indicators of more stable self-regulation and more consistent training engagement^4,23^. Independent-samples t-tests were conducted to compare anthropometric characteristics and internal load responses between groups. The analytical strategy was defined according to the repeated-measures structure of the data and the comparability of baseline values across groups. A two-way repeated-measures ANOVA (2 times × 2 groups) was used as the primary model for outcomes that did not show significant baseline between-group differences, specifically basic and psychosomatic skills, 400-m swim time, and mean velocity. This model was used to test the main effects of time and group, as well as the group × time interaction. When appropriate, post hoc comparisons were performed using Bonferroni or Games-Howell procedures depending on variance homogeneity. For cognitive skills, because a marked pre-test difference was observed between groups, an adjusted analysis was performed using ANCOVA, with post-test cognitive skills entered as the dependent variable, group as the fixed factor, and pre-test cognitive skills included as a covariate. This approach was selected to reduce the influence of baseline nonequivalence and to provide a more valid estimate of the intervention effect on cognitive skills. In addition, paired-sample t-tests were conducted within each group for cognitive skills only as supplementary descriptive analyses of pre-to-post change; these tests did not replace the primary between-group ANCOVA. Effect sizes for ANOVA and ANCOVA were reported as partial eta-squared (η²p) and interpreted as follows: small (0.01 < η²*p* < 0.06), medium (0.06 ≤ η²*p* < 0.14), and large (η²*p* ≥ 0.14)^24^. Cohen’s d was calculated to quantify the magnitude of the differences and was classified as trivial (< 0.20), small (0.20 ≤ d < 0.50), medium (0.50 ≤ d < 0.80), and large (≥ 0.80)^24^. The percentage changes from pre- to post-test (∆) were calculated for all variables. Statistical analyses were conducted using SPSS version 27 for Windows (IBM Corp, Armonk, NY, USA), and statistical significance was set at *p* ≤ 0.05.

## Results

The EG demonstrated significantly higher HRpeak and RPE values than the CG across the 12 training sessions (both *p* < 0.001, d = 3.98 and 2.71, large effects; Table 3). Despite these higher values, the intra-subject and inter-session variability of HRpeak and RPE were markedly lower in the EG (all *p* < 0.05), indicating more stable internal-load regulation. Specifically, lower CV% indicated lower dispersion in effort perception and physiological responses (Table 3).

**Table 3:** Internal load responses and variability indices (CV%) for the experimental (EG; *n* = 15) and control (CG; *n* = 15) groups.

Variable	Group	Mean	SD	95% CI	Variability (CV%)		Group comparisons		
					Intersession, 95% CI	Intrasubject, 95% CI	t	p	d
HRpeak (%HRmaxTheo)	EG	91.0	1.9	89.9–92.0	1.0-3.6	0.8–2.8	10.893†	< 0.001	3.978 (large)
	CG	85.0	0.9	84.5–85.5	1.7–5.8	2.5–9.5			
RPE (AU)	EG	6.6	0.7	6.2–6.9	5.2–18.1	3.9–17.3	7.417†	< 0.001	2.708 (large)
	CG	5.0	0.5	4.8–5.3	11.9–41.3	9.1–40.8			

The values are expressed as mean and SD with 95% confidence interval (95% CI) of the 12 training sessions performed during the intervention period by both groups. HRpeak: heart rate peak; %HRmaxTheo: percentage of the theoretical maximum heart rate; RPE: rate of perceived exertion; AU: arbitrary unit; d: cohen’s d. The intersession CV% represents the mean variability of the training load responses with the group across the intervention period and expressed as coefficient of vaiation; intrasubject CV% represents the mean variability of the training load responses of the individual subjects across the intervetion period and expressed as coefficient of variation.

†: A significant intergroup difference. The statistical significance level was set at *p* ≤ 0.05.

**Fig. 2:**
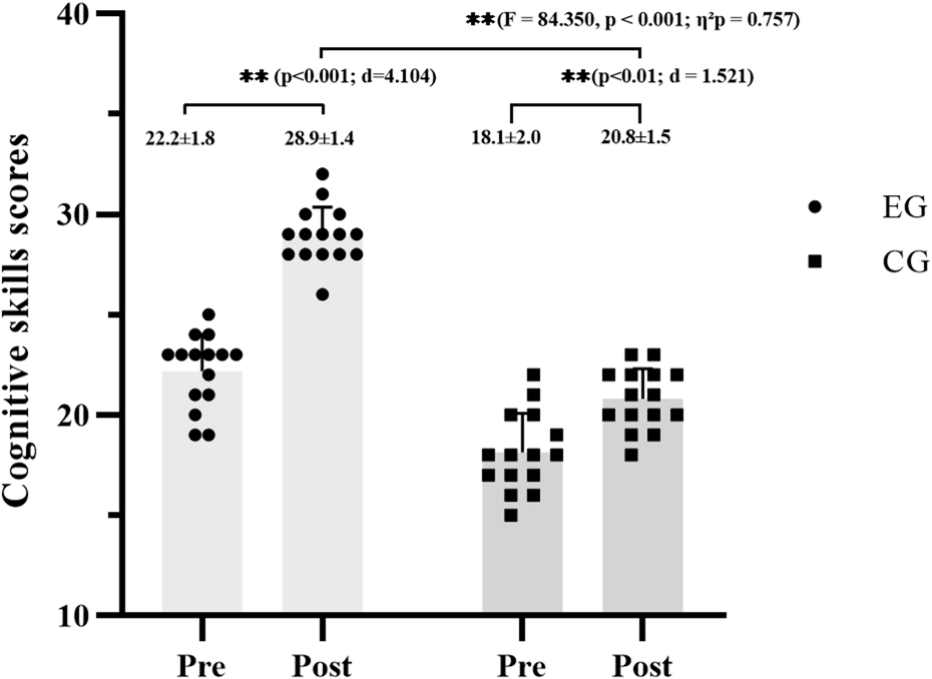
Comparison of cognitive skills’ scores between experimental group (EG; *n* = 15) and control group (CG; *n* = 15) at pre- and post-intervention.

At baseline, the two groups were statistically similar for age, height, 400-m swim time, mean velocity, and most mental skill domains (all *p* > 0.05), except for body mass (*p* = 0.026, d = 0.86, large), BMI (*p* = 0.010, d = 1.01, large), and cognitive skills (*p* < 0.001, d = 2.15, large) (Tables 1 and 4, and Fig. 2).

**Table 4:** Basic and psychosomatic skills’ scores, and endurance performance at pre- and post-intervention period for the experimental (*n* = 15) and control (*n* = 15) groups.

Variable	Group	Pre			Post			Within-group comparisons		
		Mean	SD	95% CI	Mean	SD	95% CI	Δ (%)	p	d
Basic skills	EG	12.1	1.5	11.2–12.9	16.3†	1.9	15.3–17.4	36.3 ± 16.1	< 0.001	2.454 (large)
	CG	10.9	2.1	9.8–12.1	11.9	2.5	10.5–13.2	11.7 ± 26.2	0.501	0.433 (small)
psychosomatic skills	EG	15.9	3.2	14.1–17.6	22.9†	2.2	21.7–24.1	51.3 ± 35.8	< 0.001	2.703 (large)
	CG	15.1	2.3	13.8–16.4	16.2	2.6	14.8–17.6	8.3 ± 15.8	0.177	0.448 (small)
400 m swim time (s)	EG	285.05	17.47	275.38-294.72	284.45	17.20	274.93-293.98	-0.21 ± 0.24	0.002	0.035 (trivial)
	CG	285.38	17.75	275.55-295.21	285.27	17.72	275.46-295.09	-0.04 ± 0.21	0.513	0.006 (trivial)
Mean velocity (m.s^− 1^)	EG	1.408	0.08	1.36–1.45	1.412	0.08	1.37–1.46	0.2 ± 0.2	0.011	0.040 (trivial)
	CG	1.406	0.08	1.36–1.45	1.407	0.08	1.36–1.45	0.0 ± 0.2	0.590	0.012 (trivial)

The values are expressed as mean and SD with 95% confidence interval (95% CI) in both groups. Δ : pre-to-post change percentage ; d : cohen’s d.

† A significative difference when comparing pre-test and post-test.

Significantly different from CG at post-test. The statistical significance level was set at *p* ≤ 0.05.

A significant main effect of time was found for basic skills (F = 16.14, *p* < 0.001, η²*p* = 0.37, large) and psychosomatic skills (F = 50.31, *p* < 0.001, η²*p* = 0.64, large), with both domains improving across the intervention. Significant time × group interactions were also detected for both variables (basic skills: F = 9.33, *p* = 0.005, η²*p* = 0.25; psychosomatic skills: F = 26.34, *p* < 0.001, η²*p* = 0.49, large). Post-hoc comparisons revealed that the EG achieved substantially greater gains than the CG in both basic (*p* < 0.001, d = 2.02) and psychosomatic (*p* < 0.001, d = 2.83) domains. For cognitive skills, ANCOVA adjusting for baseline differences showed higher adjusted post-test scores in the EG compared with the CG (*p* < 0.001; Fig. 2). However, CG showed no significant changes in basic or psychosomatic skills and only large improvement in cognitive skills (*p* < 0.001, d = 1.52).

A significant main effect of time was observed for 400-m swim time (F = 8.521, *p* = 0.007, η²*p* = 0.233, both large) and mean velocity (F = 5.362, *p* = 0.028, η²*p* = 0.161, both large). However, no significant time × group interactions were detected in 400 m swim time or mean velocity (F = 3.932 and 2.383, *p* = 0.057 and 0.134, η²_p_ = 123 and 0.078, both medium, respectively) nor main effects of group for these variables (F = 3.932 and 0.008, *p* = 0.057 and 0.929, η²_p_ = 0.123 and 0, medium and trivial, respectively; Table 4).

## Discussion

This study examined the effects of a three-week mindfulness program integrated into swim training on mental skills, internal-load regulation, and endurance performance in youth swimmers. The main findings were that the mindfulness intervention led to substantial improvements in mental skills (basic, psychosomatic, and cognitive domains) and greater stability in internal-load responses (reduced variability in HRpeak and RPE), whereas no significant differences emerged in 400 m performance variables between groups. These results partially confirm the hypotheses, supporting the psychological and regulatory benefits of short-term mindfulness practice but not its immediate impact on physical performance.

The most consistent changes occurred in the mental domain, where swimmers in the EG outperformed controls across all OMSAT-3 dimensions. These improvements align with previous findings indicating that structured psychological interventions can rapidly strengthen self-regulation skills such as goal setting, focus, and emotional control^1,25^. Enhancements in basic and psychosomatic skills suggest that swimmers became more capable of maintaining attentional focus and managing anxiety during training, consistent with meta-analytic evidence showing that mindfulness reduces competitive anxiety and promotes emotional stability^7,6^. Cognitive skills also improved more in the mindfulness group, echoing prior studies showing that mindfulness-based strategies, including imagery and attentional cueing, enhance decision-making and concentration under pressure^26–28^. Although the CG exhibited minor progress in cognitive skills, likely reflecting normal experiential learning, the magnitude and breadth of improvement in the mindfulness group indicate a more comprehensive enhancement of psychological functioning. In applied terms, stronger mental skills provide the cognitive foundation for consistent pacing, emotional resilience, and motivation factors that facilitate optimal training behaviors even when physical performance does not immediately change. However, the large effect sizes observed for several mental-skill outcomes should be interpreted with caution. In behavioral and sport-intervention research, effects of this magnitude are relatively uncommon and may reflect methodological influences in addition to the intervention itself. In the present study, the modest sample size may have reduced the stability of standardized estimates and increased their sensitivity to sample-specific variation. Moreover, baseline nonequivalence for some outcomes and the use of questionnaire-based measures may also have contributed to the magnitude of the observed effects. Accordingly, these values should be viewed as preliminary estimates rather than precise indicators of the true intervention effect, and they require confirmation in larger and more diverse samples.

The internal-load findings should be interpreted with caution. The mindfulness group displayed higher mean HRpeak and RPE responses together with lower variability across sessions. Although this pattern may suggest a more stable internal-load profile during the intervention period, it does not by itself demonstrate enhanced self-regulation. Indeed, higher HRpeak and RPE values may also reflect subtle differences in training execution, effort allocation, or responsiveness to the prescribed sessions rather than a specific regulatory mechanism. Mindfulness training has been associated with interoceptive awareness and attentianal control, which may influence how athletes perceive and regulate exercise demands^29,30^. Accordingly, one possible interpretation is that mindfulness contributed to more stable perceptual and physiological responses during training, but this explanation should be considered provisional rather than definitive. This interpretation aligns with evidence from acute experimental studies, where mindful attention to movement influenced perceived exertion and psychosomatic responses during exercise^31^. Therefore, lower variability in HR and RPE may be viewed as an indicator of greater response stability across sessions, but not as conclusive evidence of improved regulatory processes. Because the present analysis was based on aggregated session values and variability indices, future studies should examine session-to-session trajectories using longitudinal models in order to determine whether mindfulness alters the temporal evolution of internal-load responses during training.

No significant between-group differences were observed in 400-m freestyle performance or mean velocity, despite small within-group improvements in the EG. These null findings are consistent with the understanding that endurance performance adaptations in swimming typically require longer, periodized training cycles (≥ 6–12 weeks) encompassing technical, biomechanical, and metabolic changes^11,21,32^. Mindfulness appears to influence the psychological readiness and quality of training rather than direct physical output within a short time frame. This pattern is in line with prior systematic reviews indicating that performance-related benefits of mindfulness generally emerge after extended or repeated interventions^8,33^. Thus, while short-term mindfulness may not directly enhance race outcomes, it likely contributes indirectly by optimizing training consistency, emotional regulation, and focus, key mediators of long-term performance improvement.

From a practical standpoint, the findings demonstrate that brief mindfulness routines can be feasibly integrated into regular swim training without disrupting existing workloads. Techniques such as guided breathing, body scanning, or short pre-session meditations require minimal time yet can meaningfully enhance attentional control and perceived readiness. Coaches and sport psychologists may use variability in HR and RPE (CV%) as a sensitive indicator of response stability during training, complementing traditional load monitoring tools. Importantly, practitioners should manage expectations: in the short term, mindfulness appears to influence primarily mental and perceptual processes, whereas measurable performance gains may require longer exposure and integration into broader periodized training programs.

Several limitations should be acknowledged. First, the three-week duration of the mindfulness intervention, although intentionally brief to test its in-season feasibility, may have been insufficient to induce measurable changes in aerobic capacity or biomechanical performance. Longer interventions are likely necessary to translate psychological and regulatory improvements into observable performance gains. Second, although the sample size was supported by an a priori power analysis, the study included only 30 trained youth swimmers (15 per group) recruited from a single club. This relatively small and homogeneous sample may limit the stability of the estimates, particularly standardized effect sizes, and reduces the external validity of the findings. Third, baseline imbalances were observed for some variables, particularly body mass, BMI, and cognitive skills, indicating that randomization did not achieve full equivalence between groups. Although adjusted analyses were applied where appropriate, especially for cognitive skills, these initial differences may still have affected internal validity and should be considered when interpreting the findings. Fourth, despite rigorous assessor blinding, complete participant blinding was not possible, potentially introducing expectancy or motivational bias in self-reported psychological measures, an inherent challenge in behavioral interventions^34^. Fifth, the study relied primarily on field-based indicators of internal load (HRpeak, RPE, and their variability), which, while ecologically valid, do not fully capture underlying physiological or biomechanical mechanisms. Future research could address these limitations by implementing longer mindfulness mesocycles, recruiting larger and more diverse samples, and incorporating objective physiological (e.g., lactate) and biomechanical (e.g., stroke efficiency, pacing variability) measures. Including longitudinal follow-up assessments would also help determine the durability of mental-skill adaptations and their eventual transfer to competitive performance.

## Conclusion

This randomized controlled trial shows that integrating a short-term mindfulness program into swim training can significantly improve mental skills and promote a more stable internal-load profile in youth swimmers. These adaptations reflect primarily psychological and perceptual benefits rather than immediate performance gains. Mindfulness should therefore be viewed as a supportive training strategy that strengthens athletes’ mental readiness and internal awareness during demanding training phases. Whether these benefits translate into long-term performance improvement remains to be established.

## Supplementary Information

Below is the link to the electronic supplementary material.

Supplementary Material 1

Supplementary Material 2

Supplementary Material 3

## Data Availability

Data supporting the findings of this study are available from the corresponding author upon reasonable request.
