# Supplementary material for: A three-week mindfulness intervention on mental skills, internal-load regulation, and performance in youth swimmers: a randomized controlled trial
Source: Sci Rep. 2026 Apr 14;16:17448. doi: 10.1038/s41598-026-48457-8 (PMC13236989; doi:10.1038/s41598-026-48457-8)
Supplement: Supplementary file 2 — Supplementary Material 2 [file 41598_2026_48457_MOESM2_ESM.pdf]

TRIAL PROTOCOL

Effects of a Three-Week Mindfulness Intervention on Mental Skills, Internal-Load Regulation, and Performance in Youth Swimmers: A Randomized Controlled Trial

Project summary

This protocol describes a parallel-group, randomized controlled trial evaluating whether integrating a brief mindfulness program into routine swim training improves mental skills and stabilizes internal-load responses in trained youth swimmers. Thirty swimmers (20 males, 10 females; mean age approximately 19-20 years) from a single club are allocated 1:1 to an experimental group (EG; mindfulness + standard swim training; n=15) or a control group (CG; standard training only; n=15). Randomization uses a computer-generated permuted-block sequence stratified by baseline 400 m freestyle performance, with allocation concealment until after baseline testing and participant coding. The intervention lasts 3 weeks and includes 12 mindfulness sessions (4 sessions/week) delivered before each swimming session in EG. Both groups complete the same in-season swim microcycle (four weekly sessions of ~65-95 minutes, with no competitions during the intervention), matched for overall volume and intensity (reported range ~65-95% of theoretical HRmax). Primary outcomes are mental skills assessed with OMSAT-3 (basic, psychosomatic/affective, and cognitive domains) and endurance performance in a 400 m front crawl time trial (time and mean velocity), measured pre- and post-intervention. Internal load is monitored across all training sessions using peak heart rate (HRpeak; Polar Team Pro) expressed as % of theoretical HRmax and rating of perceived exertion (RPE; Borg CR10) collected 3 minutes post-session. Assessments are conducted under standardized conditions at the club pool during habitual evening hours, with familiarization sessions in the week before baseline testing. Safety monitoring consists of injury/illness surveillance during training and testing, with medical referral and withdrawal from activity as required. The primary hypothesis is that mindfulness training will improve mental skills and reduce variability in internal-load responses, with limited short-term changes expected in 400 m performance over a 3-week period.

General information

- Protocol title: Effects of a Three-Week Mindfulness Intervention on Mental Skills, Internal-Load Regulation, and Performance in Youth Swimmers
- Study type: Two-arm randomized controlled trial with repeated measures (pre-post).
- Protocol identifying number: Ethics approval number: 013/2020; approval date: 9 December 2020.
- Study period (as implemented): In-season period January to March 2021 (5 weeks total: 1 week baseline testing, 3 weeks intervention, 1 week post-testing).
- Trial registration: Not applicable (this sport-training study was not registered in a clinical trial registry; therefore, no registration number or First Posted date is available).
- Sponsor/Funder: None (no external funding).
- Coordinating investigators (corresponding author): John Elvis Hagan, Professor PhD; Email: elvis.hagan@uni-bielefeld.de ; Vlad Adrian Geantă, Assistant Professor PhD; vlad.geanta@uav.ro

- Investigators: Mohamed Ali Sifi; Hamza Marzouki; Okba Selmi; Bilel Sdira; Bilel Cherni; John Elvis Hagan; Dan Iulian Alexe; Alexandru Ioan Băltean; Vlad Adrian Geantă; Wafa Douzi; Anissa Bouassida.
- Research site(s): Single club setting; 25 m pool (depth 1.50-2.50 m). Testing and training conducted in the evening (approximately 17:30-20:30) under standardized pool (28-29°C) and ambient (27-28°C) temperatures.
- Clinical laboratories/technical departments: Not applicable (field-based and pool-based testing only).

Rationale and background

Mindfulness-based approaches are used in sport to enhance attention control, emotion regulation, and self-awareness, which are central to self-regulation and consistent training execution. Swimming is a closed-skill, pacing-dependent sport performed in a sensory-restricted environment; therefore, athletes rely heavily on interoceptive awareness to manage effort, breathing constraints, and technique over sustained distances. Monitoring heart rate and perceived exertion across repeated sessions provides a practical window into internal-load regulation and training consistency. Although mindfulness interventions in sport have reported improvements in mental skills and stress regulation, evidence in aquatic disciplines remains limited and performance effects appear sensitive to context and intervention duration. This protocol tests the feasibility and short-term effects of a brief (3-week) integrated mindfulness program delivered immediately before swim training, with a focus on mental skills and stability (variability) of internal load.

Study objectives

To determine whether a 3-week (12-session) mindfulness program integrated before swim training improves mental skills (OMSAT-3) and modifies internal-load regulation (HRpeak and RPE level and variability) compared with standard training alone, and to evaluate whether these changes transfer to short-term endurance performance in a 400 m front crawl time trial (time and mean velocity).

Study design

- Design: Parallel-group randomized controlled trial with pre- and post-intervention testing.
- Allocation ratio: 1:1 (Experimental Group vs Control Group).
- Total duration per participant: 5 weeks (Week -1 familiarization; Week 0 baseline testing; Weeks 1-3 intervention; Week 4 post-testing).
- Blinding: Outcome evaluation supported by participant coding; assessors not involved in training/testing manage codes. Participants and coaches cannot be blinded due to the nature of the intervention.
- Setting: In-season club training microcycle of four weekly sessions; no competitions during intervention.

Study population and eligibility

- Population: Trained youth swimmers from a single club; regularly competing at the national level.

- Sample size: n=30 planned and recruited (EG n=15; CG n=15). Sample size estimated a priori (minimum n=24) using repeated-measures ANOVA within-between interaction assumptions (alpha=0.05, power=0.80, f=0.30).
- Inclusion criteria: At least 2 years of continuous swim training; no severe musculoskeletal injury in the past year; no mild/moderate injury in the past month; no prior experience with mindfulness programs.
- Exclusion criteria: Does not meet inclusion criteria; injury/illness preventing safe participation; prior mindfulness experience.
- Retention / analysis set: Only participants completing at least 90% of all training and testing sessions are included in the final analysis.
- Withdrawal criteria: Participant request; medical advice; injury/illness preventing safe continuation; non-adherence (<90% sessions).

Methodology

- Recruitment and consent**:** Participants are recruited from a single club. Written informed consent is obtained from all participants; for minors, parental consent is also secured. Participants are asked to maintain usual diet and avoid additional fitness/dietary practices during the study.
- Randomization and allocation concealment**:** Randomization is performed by an independent researcher using a computer-generated sequence with permuted blocks stratified by baseline 400 m freestyle performance. Allocation is concealed until after baseline testing. Following baseline assessments, participant identifiers are coded; code management is handled by a blinded assessor not involved in testing or training. Group assignments are communicated only after enrollment is completed.
- Blinding**:** Due to the behavioral nature of the intervention, participants and coaches are not blinded. To reduce assessment bias, participants are coded and managed by an assessor not involved in training. Outcomes are collected under standardized procedures with identical instructions and timing for both groups.
- Familiarization: One week prior to baseline testing, all participants complete two familiarization sessions approximately 48 hours apart. Familiarization includes instruction on testing procedures, acclimatization to the heart-rate monitoring system (Polar Team Pro), and standardized use of Borg RPE (0-10). Anthropometrics (body mass, height; BMI calculated) are recorded during this phase.
- Interventions**:** Both groups follow the club's in-season swim microcycle of four weekly sessions (~65-95 minutes each), with no competitions during the intervention. Training intensity is monitored and described as approximately 65-95% of theoretical HRmax. The key difference between groups is the addition of a pre-session mindfulness component in the experimental group.
    - **Experimental group (EG):** Receives a 30-45 minute mindfulness session immediately before each swim session, for a total of 12 sessions over 3 weeks (4 sessions/week). The mindfulness component is adapted from evidence-based protocols and delivered by a certified mental training instructor independent of the research team to reduce expectancy bias. Core techniques include diaphragmatic breathing, box breathing, body-scan meditation, and present-moment awareness exercises. Attendance is recorded.

- **Control group (CG):** Completes standard swim routines and skill-development activities consistent with usual training practices. No formal mindfulness sessions are provided.
- **Co-interventions:** Participants maintain usual training, sleep, and diet routines and refrain from additional fitness/dietary practices. No competitions occur during the intervention as described in the manuscript.

Outcome measures and testing procedures

- Mental skills: OMSAT-3 (Arabic validated version); 12 mental skills grouped into three domains: basic (goal setting, self-confidence, commitment), psychosomatic/affective (stress reactions, fear control, relaxation, activation), and cognitive (imagery, mental practice, control of distractions, competition planning, self-assessment). Administered 30 minutes before a training session on the second testing evening.
- Endurance performance: 400 m front crawl maximal time trial (time in seconds and mean velocity). Performed after a standardized warm-up; paired by skill level; race order randomized.
- Internal load: HRpeak continuously monitored during each session using Polar Team Pro; recovery periods excluded; expressed as % of theoretical HRmax (HRmaxTheo = 208 - 0.7 × age). RPE collected 3 minutes post-session using Borg CR10 (0-10 AU). Variability across sessions quantified as coefficients of variation (CV%) at inter-session and intra-subject levels.

Standardization and equipment (summary)

- Timing: Tests conducted during habitual evening hours (approximately 17:30-20:30) and scheduled ~48 hours after the last competition or high-intensity session to reduce fatigue confounding.
- Warm-up: Standardized warm-up for the 400 m test: ~1000 m low-to-moderate intensity freestyle selected by swimmers, followed immediately by the 400 m maximal effort.
- Performance timing: 400 m time recorded by two independent observers using SEIKO S120-4030 stopwatches; mean of the two times used.
- Setting control: Pool and ambient temperatures standardized (pool 28-29°C; ambient 27-28°C).

Schedule of assessments:

Phase	Timing	Assessments	Notes
Familiarization	Week -1 (2 sessions, ~48 h apart)	Test instructions; HR monitor familiarization; Borg RPE instruction; anthropometrics	Conducted before baseline testing
Baseline	Week 0 (two evenings)	400 m front crawl; OMSAT-3	Testing scheduled ~48 h after last high-intensity session; identical procedures for both groups
Intervention	Weeks 1-3 (4 sessions/week; 12 sessions total)	EG: mindfulness (30-45 min) + swim training; CG: swim training only; HRpeak and RPE each session	Training sessions 65-95 min; intensity monitored (~65-95% HRmaxTheo)

Post-test	Week 4 (two evenings)	Repeat 400 m front crawl; OMSAT-3	Same procedures and timing as baseline

Participant flow diagram

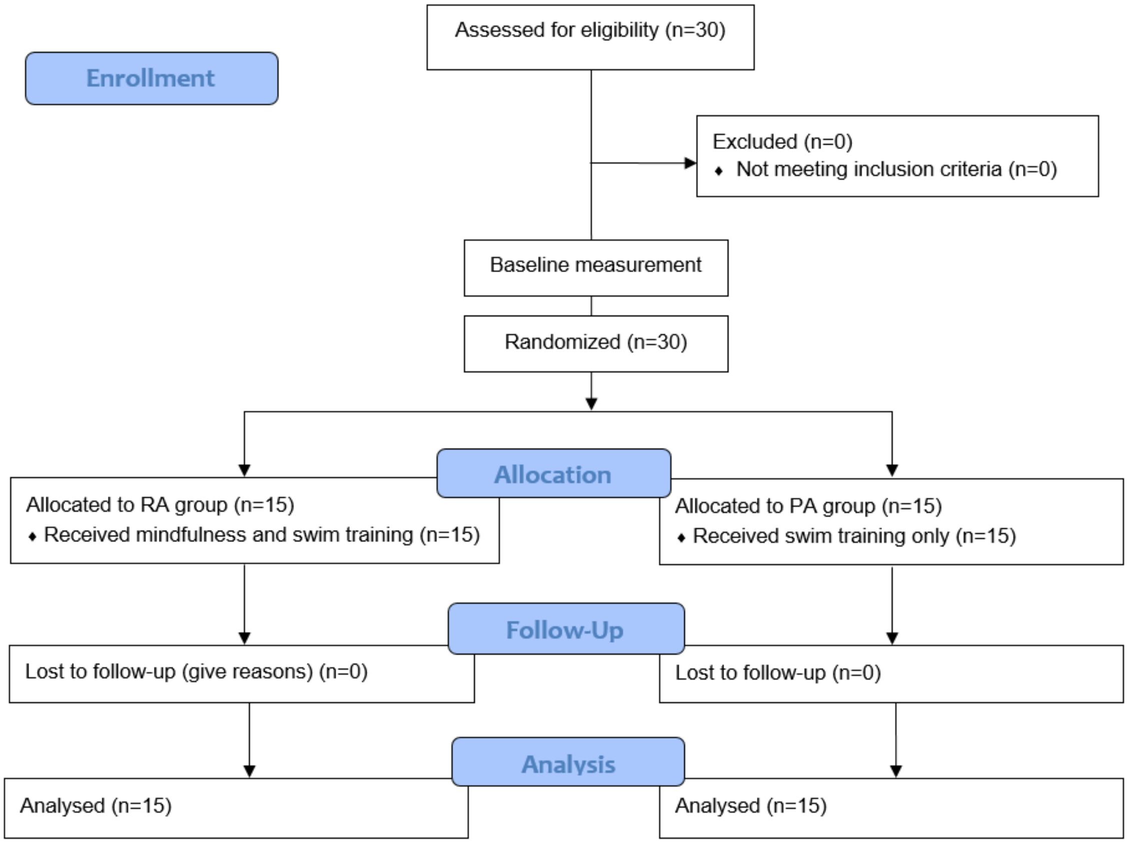

Safety considerations

Risks include acute musculoskeletal injury during training and testing and adverse responses to maximal efforts. Risk mitigation includes pre-participation screening for recent injury, standardized warm-ups, progressive training design, qualified supervision, and cool-down after maximal tests. Injuries, illness, and any untoward events are monitored throughout the study; participants are withheld from training/testing when needed and referred to medical care as appropriate.

Follow-up

Participants are monitored across the 5-week study for attendance, internal-load responses (HRpeak and RPE each session), and adverse events. Follow-up ends after post-testing; no long-term follow-up beyond the post-intervention assessment week is described.

Data management and statistical analysis

- Data capture: Session HR and RPE data are recorded during each training session; performance and questionnaire outcomes are recorded during pre/post testing.
- Confidentiality: Participants are assigned coded identifiers; the code key is managed separately by an assessor not involved in training/testing.
- Primary analysis: Two-way repeated measures ANOVA (2 times × 2 groups) for main and interaction effects; alpha set at $p \leq 0.05$.
- Additional analyses: Normality checked with Shapiro-Wilk; homogeneity with Levene. Non-normal variables log-transformed. Where baseline differences exist,

ANCOVA with pre-test covariates is applied (noted for cognitive skills). Post hoc comparisons use Bonferroni or Games-Howell depending on variance assumptions.
- Effect sizes: Partial eta-squared for ANOVA/ANCOVA (small 0.01-0.06; medium 0.06-0.14; large ≥0.14) and Cohen's d for between-group differences.
- Variability: Intra-subject and inter-session variability of HRpeak and RPE computed as coefficients of variation (CV%) with 95% confidence intervals.
- Software: SPSS v27 for Windows (IBM).

Quality assurance
Procedures are standardized across groups and time points, including testing order, warm-up, equipment, and environmental conditions. Familiarization sessions are used to reduce learning effects. Mindfulness sessions are delivered by an instructor independent of the research team. Training attendance is recorded to verify adherence (≥90% threshold).

Expected outcomes of the study
Mindfulness is expected to improve mental skills (basic, psychosomatic/affective, and cognitive domains) and enhance internal-load regulation evidenced by reduced variability across sessions. Given the short duration, large between-group changes in 400 m performance are not necessarily expected.

Dissemination of results and publication policy
Results will be disseminated through peer-reviewed publication(s) and scientific presentations. Authorship and contributor acknowledgment should follow standard journal policies (e.g., ICMJE criteria), as indicated by the manuscript's authorship contribution section.

Duration of the project
Overall duration per participant: 5 weeks (including familiarization, baseline, intervention, and post-testing).

Week	Activities	Outputs
Week -1	Familiarization (2 sessions); anthropometrics; HR and RPE familiarization	Participant list and codes; baseline readiness
Week 0	Baseline testing (400 m front crawl; OMSAT-3)	Baseline dataset
Weeks 1-3	Intervention (12 sessions total); HRpeak and RPE recorded each session	Training adherence log; internal-load dataset
Week 4	Post-testing (repeat 400 m and OMSAT-3)	Post-test dataset; finalized trial dataset
Post-study	Data cleaning, analysis, and manuscript preparation	Statistical outputs; manuscript draft

Problems anticipated
Potential issues include missed sessions due to illness/injury, variable motivation, and day-to-day environmental variation. Mitigation includes standardized testing conditions and timing, familiarization to reduce measurement noise, attendance tracking with a predefined adherence threshold, and consistent monitoring of internal load using both physiological (HR) and perceptual (RPE) indicators.

Project management

The coordinating investigator oversees trial conduct. An independent researcher generates the randomization schedule and releases allocation after baseline testing. A blinded assessor manages coded identifiers. The mindfulness component is delivered by a certified instructor independent of the research team. Statistical analyses are performed according to a predefined plan using standard software.

Ethics

The study is conducted in accordance with the Declaration of Helsinki and approved by the institutional research ethics committee of the Higher Institute of Sport and Physical Education of Kef, University of Jendouba, Tunisia (approval number: 013/2020; approval date: 9 December 2020).

Informed consent forms

Written informed consent is obtained from all participants before any study procedures. For minors, parental consent is also obtained. Consent documents cover purpose, procedures, risks/benefits, confidentiality, voluntary participation, and investigator contact details. Signed forms are retained securely by the investigators.
