# Supplementary material for: A three-week mindfulness intervention on mental skills, internal-load regulation, and performance in youth swimmers: a randomized controlled trial
Source: Sci Rep. 2026 Apr 14;16:17448. doi: 10.1038/s41598-026-48457-8 (PMC13236989; doi:10.1038/s41598-026-48457-8)
Supplement: Supplementary file 3 — Supplementary Material 3 [file 41598_2026_48457_MOESM3_ESM.docx]

Project: Effect of combined mindfulness and swimming training program on performance, physiological responses, and mental skills in young swimmers.

**Parental/Legal gueardian and partcicipant consent to take part in research**

**Responsible researcher:** Mohamed Ali Sifi

**Institution:** High Institute of Sport and Physical Education of Ksar Saïd, University of Manouba, Tunisia.

**Ethic Committee Name:** Research Ethics Committee of the High Institute of Sport and Physical Education of Kef (UR22JS01), El Kef, University of Jendouba, Tunisia.

**Approval Code:** 013/2020

**Approval Date:** December 9, 2020

**Phone:** +21622379846

____________________________________________________________________________

This consent form aiming to explain that, after reading the respective document, I agree to participate in the study '' Effect of combined mindfulness and swimming training program on performance, physiological responses, and mental skills in young swimmers. ''

**PROCEDURES**: I have been informed that the general objective will be " to assess whether incorporating mindfulness into swimming training (3 weeks, frice a week) enhances physical fitness, physiological responses, and mental skills in young swimmers''. The study will be conducted during the in-season period from January to March 2021, and will last five weeks. The first and last weeks will focus on measuring my 400m crawl swim time and mental skills (using the Arabic version of the Ottawa Mental Skills Assessment Tool). The third, fourth, and fifth weeks will be dedicated to performing a combined mindfulness and swimming training program. A qualified mental instructor will lead the mindfulness program before the swimming component. My heart rate will be continuously recorded throughout each training session using heart rate monitors. After each training session, I will rate my effort using Borg scale. The results will be kept confidential and used for scientific research purposes.

**RISKS AND POSSIBLE REACTIONS**: I was informed that the risks are minimal, because I was screened by an experienced physician and was found eligible to participate in the study. In the event of any more serious injury, a doctor who is specialist in sports medicine will be immediately communicated to make appropriate measures.

**BENEFITS**: The benefit of participating in the research is related to the fact that the results will be incorporated into scientific knowledge and later to the practical applications of the effect of a combined mindfulness and swimming training program on endurance performance, physiological responses, and mental skills in young swimmers.

**VOLUNTARY PARTICIPATION**: As I have been told, my participation in this study will be voluntary and I may stop participating at any time.

**EXPENSES**: I will not have to pay for any of the procedures, nor will I receive financial compensation.

**CONFIDENTIALITY**: I am aware that my identity will remain confidential during all stages of the study.

**CONSENT**: I received clear explanations about the study, all recorded in this consent form. The study researchers answered and will answer, at any stage of the study, all my questions, until my complete satisfaction. Therefore, I agree to participate in the study. This Pre-Informed Consent Form will be signed by me and filed with the institution responsible for the research.

**INVESTIGATOR'S STATEMENT OF RESPONSIBILITY:** I explained the nature, objectives, risks, and benefits of this study. I made myself available for questions and answered them in their entirety. The participant understood my explanation and accepted, without impositions, to sign this consent. I am committed to using the data and the material collected for the publication of reports and scientific articles related to this research.

Participant name/Parental or legal guardian: _____

Identity: __________

SIGNATURE: ________

SIGNATURE OF THE RESPONSIBLE RESEARCHER: _________
